# Double-Needle Arthrocentesis with Viscosupplementation in Patients with Temporomandibular Joint Disc Displacement without Reduction

**DOI:** 10.6061/clinics/2021/e2840

**Published:** 2021-04-16

**Authors:** Rafael Rossini, Eduardo Grossmann, Rodrigo L. Poluha, Ênio T. Setogutti, Marcos Fabio Dos Santos

**Affiliations:** I Pos-Graduacao em Ciencias Cirurgicas, Universidade Federal do Rio Grande do Sul, Porto Alegre, RS, BR.; II Departamento de Ciencias Morfologicas, Universidade Federal do Rio Grande do Sul, Porto Alegre, RS, BR.; III Departamento de Protese, Universidade de Sao Paulo, Bauru, SP, BR.; IV Clinica Particular, Porto Alegre, RS, BR.; V Instituto de Ciencias Biomedicas, Universidade Federal do Rio de Janeiro, Rio de Janeiro, RJ, BR.

**Keywords:** Oral Surgery, Magnetic Resonance Imaging, Arthrocentesis, Temporomandibular Joint, Viscosupplementation

## Abstract

**OBJECTIVES:**

Arthrocentesis is the simplest surgical intervention for the temporomandibular joint (TMJ). It can be performed on an outpatient basis at a low cost and with low morbidity. The objective is to release the articular disc by disrupting the adhesion formed between its surfaces and the mandibular fossa through hydraulic pressure generated by irrigation of the upper compartment of the TMJ. Viscosupplementation with hyaluronic acid during or after arthrocentesis improves clinical outcomes, increases mouth opening, and reduces pain levels. The aim of this study was to evaluate the efficiency of arthrocentesis plus hyaluronic acid viscosupplementation through clinical examination and preoperative magnetic resonance imaging in patients with unilateral disc displacement without reduction (DDwoR).

**METHODS:**

This analytical retrospective cross-sectional study clinically and radiologically evaluated 72 patients of both sexes with unilateral DDwoR. The following data were collected: sex, pain, age, duration of pain, maximum mouth opening, and patient pain perception on a visual analog scale. TMJ arthrocentesis was performed only once for each of the indicated joints. Data were collected before arthrocentesis (baseline) and at 7, 14, 30, 60, 90, and 180 days after the procedure (final evaluation).

**RESULTS:**

Between the baseline and final evaluation, there was a significant reduction in pain (*p*=0.001) and restoration of articular function. In addition, there was a significant increase in maximum mouth opening (*p*=0.001).

**CONCLUSION:**

Patients with DDwoR undergoing arthrocentesis combined with hyaluronic acid injection showed significant improvement in the perceived pain and maximum mouth opening in the mid-term follow-up periods.

## INTRODUCTION

Temporomandibular disorder (TMD) is an umbrella term that covers all abnormalities of the masticatory muscles, temporomandibular joint (TMJ), and accessory structures (1). Clinical symptoms of temporomandibular disorder include local pain in the TMJ, limited mouth opening, noise, and joint clicking. It is a common disease, currently estimated to affect approximately 5% of the American adult population, with a female predominance (2,3).

Internal disorders of the TMJ are characterized by an abnormal relationship between the mandibular condyle, tubercle, and articular disc (4), as well as articular degeneration, such as osteoarthritis and osteoarthrosis. From this perspective, several strategies, including physiotherapy, pharmaceutical agents, and clinical treatments, have been employed to reduce pain and improve the range of jaw motion (5). When there is no effective response after 3 months of conservative treatment, a surgical alternative, such as arthrocentesis, may be necessary (3,6).

This surgical procedure was first described by Nitzan et al. (7). Exceptional results have been achieved by employing arthrocentesis of the upper compartment of the TMJ, including restoration of mouth opening to a normal level and relief of pain (7,8). This procedure is considered minimally invasive and can be performed under local anesthesia with low morbidity (9).

The aim of arthrocentesis is to remove inflammatory mediators and cellular debris, and release adhesions between the disc and articular fossa by washing out the upper compartment of the TMJ with simultaneous manipulation of the jaw (10). In addition to patients with limited mouth opening (8,11,12), arthrocentesis is also indicated for cases of disc displacement with and without reduction (DDwoR) (11-15), synovitis/capsulitis, rheumatoid arthritis, and osteoarthritis (16,17). Considering the low risk of adverse events and the ability to yield faster and durable relief of pain, some authors (18,19) suggest injecting sodium hyaluronate after arthrocentesis. Studies have shown that this combination provides greater patient comfort and longer-lasting results (16,20,21).

Hyaluronic acid (HA) is a natural hydrophilic mucopolysaccharide glycosaminoglycan, and a component of both synovial fluid and cartilage tissue. This fluid combines with glycosaminoglycans to form proteoglycans, which disintegrate and disperse in the synovial cavity under pathological conditions. Although its half-life is only 13h, HA demonstrates beneficial effects when administered by intraarticular injection (18,22).

Elucidation of the advantages of arthrocentesis with HA viscosupplementation in patients with DDwoR will help oral and maxillofacial surgeons indicate or employ this technique in a more consistent and qualified manner, thereby offering, a simpler and more effective treatment to patients suffering from this disease.

## MATERIAL AND METHODS

This was an analytical, retrospective, and cross-sectional study. This study was approved by the Research Ethics Committee of the Federal University of Rio Grande do Sul (CAAE: 59616416.8.0000.5347) and was conducted in accordance with the Declaration of Helsinki and the Consolidated Standards of Reporting Trials (CONSORT) statement. All patients provided written informed consent for participation.

For 90% statistical power and a 5% level of significance, the sample size was calculated as 34 subjects (68 observations) to observe reduction in pain and reestablishment of the maximum mouth opening (MMO).

Adult individuals (age ≥18 years) of either sex, diagnosed with DDwoR causing unilateral TMJ pain that did not respond to ≥3 months of conservative treatment (e.g., interocclusal appliances, anti-inflammatory drugs, a soft diet, and physiotherapy) were deemed eligible.

The diagnosis of DDwoR was confirmed by clinical examination, based on axis I of the Research Diagnostic Criteria for Temporomandibular Disorder (23-25), and magnetic resonance imaging (MRI) findings, as described by Ahmad et al. (26). All MRI scans were interpreted and reported by the same radiologist.

Patients with rheumatoid arthritis, agenesis, hyperplasia, hypoplasia, and/or malignant neoplasm of the mandibular condyle, bony ankyloses, myopathies, a history of previous TMJ surgery or arthrocentesis (alone or with administration of other substances), and extreme needle phobia, were ineligible for participation.

In addition, we excluded patients who had disc displacement with reduction, systemic inflammatory diseases of the joints; a history of trauma to the chin or other facial bones, cardiovascular or hematological diseases, chronic use of medications such as anticoagulants or antihypertensives, neuropathic pain syndromes such as trigeminal neuralgia (especially involving the third branch), neuralgia of the intermedius, glossopharyngeal, or upper laryngeal nerves, Eagle syndrome, Ernest syndrome, confirmed or suspected pregnancy, and any relative or absolute contraindications to MRI (such as a history of claustrophobia or permanent makeup/tattoos performed <3 months before examination), as well as those currently taking analgesics or anti-inflammatory agents. Patients who were self-medicating with the latter were accepted if they completed a washout period of at least 1 week before evaluation.

All patients were evaluated, diagnosed, and treated between March 2014 and July 2016 at the Center for Orofacial Pain and Deformity (CENDDOR), Porto Alegre, Rio Grande do Sul, Brazil. The research was led by a doctor of dental surgery (EG) who was familiar with the evaluation protocol. The following data were collected: sex, age, duration of pain, in months, maximum mouth opening (MMO) in millimeters, measured with a digital caliper (Vonder^®^; range, 150 mm), presence of effusion at baseline, and perceived pain on a visual analog scale (VAS). MMO and VAS score were obtained before arthrocentesis (baseline), and at 7, 14, 30, 60, 90, and 180 days after the procedure (final).

### MRI

MRI was performed using a 1.5-Tesla (T) magnetic field scanner (General Electric Signa HDX) at Serviço de Investigação Diagnóstica, an independent imaging center. T1-weighted (TR=567 ms, TE=11.4 ms) and T2-weighted (TR=5200 ms, TE=168.5 ms) sequences were performed using a 9-cm diameter bilateral surface coil. The matrix employed for T1 was 288x192, 3 NEX, and for T2, 288x160, 4 NEX, with a field of view of 110x110 mm. Six oblique sagittal slices (3 mm thickness, 10% spacing) perpendicular to the axis of the mandibular condyle were obtained for each TMJ, at maximum intercuspation and MMO. Before axial slices were obtained, a sagittal scout view was acquired for the localization of the mandibular condyle. Six slices of each TMJ were obtained in the oblique coronal plane (T1, T2), parallel to the axis of the mandibular condyle, all in habitual occlusion (maximum intercuspation). To keep the patient relaxed, minimize movement, and maintain MMO, an occlusal splint was placed in the interincisal space. The mean scan duration was approximately 30 min. Twelve 3x4 images at 1.5x magnification were documented on a 43x35 cm film.

All the MRI films were read and interpreted by an experienced radiologist. The evaluation protocols and MRI reports were reviewed and transcribed into a Microsoft Excel spreadsheet (Microsoft Corporation).

### Double-Needle Arthrocentesis with HA Viscosupplementation

TMJ arthrocentesis was performed only once for each of the indicated joints. The procedure was performed following the technical protocols reported in the literature (7,27,28). First, patients were placed supine in a dental chair and asked to rotate their head to the asymptomatic side. The head was wrapped in a disposable cap, which was secured with Micropore^®^ tape, leaving only the TMJ area exposed. A surgical site marker was used to draw a straight line from the midpoint of the tragus to the corner of the eye (canthal-tragus distance), and two points were marked on this line for needle insertion: the first and most posterior point, 10 mm anterior to the tragus and 2 mm below the cantho-tragal line; the second, 20 mm anterior to the tragus and 10 mm inferior to the cantho-tragal line. Antisepsis was performed with 2% chlorhexidine solution, which was applied liberally to the entire face, mainly in the preauricular area and pinna. The next step was an auriculotemporal nerve block (2% lidocaine hydrochloride without vasoconstrictor, 1.8 mL), followed by anesthesia of the posterior deep temporal and masseteric nerves (one or two tubes). The objective of analgesia was to prevent any discomfort and/or pain during the procedure, thus rendering sedation unnecessary. The patient was then requested to open the mouth as far as possible, effecting downward and forward displacement of the condyle, which enabled access to the posterior recess of the upper compartment of the TMJ, where the first needle (40x1.2 mm, 18G) was introduced. The needle was directed anteriorly, superiorly, and medially until its tip hit the mandibular fossa within the upper compartment. The needle was then connected to a 5-mL syringe, and 4 mL of 0.9% saline solution was injected to distend the joint space. Next, the syringe was removed and the needle was connected to a 100-cm long clear plastic extender (Compojet^®^, Compojet Biomédica LTDA, Conceição do Jacuípe, BA, Brazil). A second needle, with the same dimensions as the first needle, was introduced into the distended compartment, at the prior point of entry, and connected to a 60-cm long, 20G flexible clear catheter (Mark Med^®^, Mark Med LTDA, Bragança Paulista, SP, Brazil) that was used to visualize the flow of the articular washout. The other extremity of this catheter was connected to a rubber suction tip (DabiAtlante^®^, DabiAtlante LTDA, Ribeirão Preto, SP, Brazil), which was driven by a vacuum pump (PVD700-4C/DabiAtlante^®^, DabiAtlante LTDA, Ribeirão Preto, SP, Brazil).

Using 60 mL syringes, 200 mL of 0.9% saline solution was injected through the extender connected to the first needle and withdrawn through the cannula connected to the second needle. No other substances or drugs were added to the injected solution. The cannula connected to the second needle was occluded for approximately 10s while injecting the last 5 mL of saline, increasing the hydraulic pressure within the upper compartment of the TMJ. The patient was then instructed to open his or her mouth and move the mandible laterally to break up any possible adhesions, thus allowing gain of vertical and lateral range of motion, which was measured intraoperatively. After arthrocentesis was completed, the second needle was removed, and 1 mL of HA solution (sodium hyaluronate, 1,000-2,000 kDa) was injected. The needle was removed, and the puncture sites were covered with a spot bandage (Cremer Ltda., São Paulo, SP, Brazil) that was left in place for 1h.

 Patients were instructed to take 750 mg paracetamol every 6h for 3 days, intermittently apply ice locally for the first 48h, consume only liquids and pureed foods for 5 days, wear a thermoplastic maxillary occlusal splint (with simultaneous bilateral contact with the mandibular teeth) for the same period, removing it only during meals and for oral hygiene, and avoid physical activities and local heat application for 1 week.

### Statistical Analysis

Categorical variables (patient gender and size, effusion, and position of the disc) were analyzed using the chi-square test and are shown as numbers and percentages. The variable with normal distribution (age) was analyzed using Student’s t-test and is presented as the mean and standard deviation. The variable with asymmetric distribution (pain duration) was assessed using the nonparametric Mann-Whitney test and is presented as the median and interquartile range. To observe the efficacy of the therapy, we compared the MMO values before and after the procedure. To evaluate test tolerance, pain perception before and after surgical intervention was assessed. In these cases, the nonparametric Wilcoxon test was used for analysis.

All analyses were performed using PASW Statistics for Windows (Version 18.0; SPSS Inc., Chicago, Illinois, USA), at a significance level of 5% (*p*<0.05).

## RESULTS

The 72 patients were evaluated over a 6-month period after arthrocentesis. There were no losses or withdrawals, and no complications were observed during or after the procedures.

Data for the overall sample (Table 1) showed a higher prevalence of DDwoR in female subjects. In addition, most patients had an effusion in the upper compartment and anterior disc displacement, with no lateral or medial components. The variables painful side and deflection were distributed homogeneously.

Table 2 shows the average age, MMO and pain at baseline, the duration of pain and changes from baseline in MMO and pain after surgical intervention (ΔMMO and ΔPain). There was substantial recovery of function (increased MMO) after arthrocentesis, as well as a significant reduction in pain.

Both MMO and pain improved in response to therapy, that is, arthrocentesis with viscosupplementation resulted in resolution of pain and improvement in MMO (Table 3).

It can be inferred that the greater the final MMO, the lower the final pain score. Effusion in the upper compartment correlated moderately with MMO (*p*=0.003). However, this correlation was strong when effusion was present in both compartments (*p*<0.001). This suggests that the study intervention effectively improved both variables (*p*<0.001) (Table 4).

## DISCUSSION

Arthrocentesis has been proposed as an effective approach for the treatment of patients with DDwoR. Beneficial effects on mandibular mobility are well established, and may be attributed to the removal of adhesions, reduction, or elimination of negative pressure within the joint, distension of the joint space, and modification of the viscosity of the synovial fluid, thus facilitating movement of the articular disc and condyle (7,10,28).

Effusions are defined as large collections of synovial fluid in the joint space and are associated with DDwoR (29). The findings of this study indicate that the location of the effusion may be relevant in patients with DDwoR, and that this parameter may predict a better treatment response in terms of reduction of pain and increase in MMO. Even if a positive association exists between effusion and DDwoR, the former should not be interpreted as a causal agent of the latter. Further observations, such as MRI showing disappearance of or decrease in effusion, are necessary to confirm this assumption. In addition, inflammation and subsequent pain may also be considered an adaptation of the body to the displaced disc to increase joint lubrication and reduce friction.

The primary reason why patients with TMJ disorders seek treatment is pain (2). Early treatment of DDwoR with conservative methods or arthrocentesis is beneficial; however, the latter appears to be superior in terms of pain relief (3,8,20). In the present study, there was a statistically significant reduction (*p*=0.001) in pain perception after arthrocentesis. This was expected because the abundant irrigation with biocompatible substances allows removal of debris from the degenerating joint tissues, eliminating algogenic substances and, especially, inflammatory mediators (16). In addition, sodium hyaluronate has analgesic and anti-inflammatory properties (20,22).

The literature suggests that adequate pain control during arthrocentesis facilitates maintenance of the needles in their proper position and decreases painful stimuli to the central nervous system (12), in addition to improving the patient’s comfort and confidence to perform the requested mandibular movements, regardless of the arthrocentesis technique employed. We performed anesthetic blockade of the auriculotemporal, posterior deep temporal, and masseteric nerves, which may have contributed to the reduction in pain and increase in MMO.

The positive results of the technique used in this study may also be due to the administration of HA immediately after arthrocentesis. The authors (16,22) have shown that results tend to be better when this combination (HA and arthrocentesis) is applied than when only arthrocentesis is performed. In fact, saline solution is reported to contribute mainly to the primary effect after the operation, while HA is responsible for the subsequent maintenance of the initial results (12).

HA promotes increased joint lubrication by increasing the viscosity of synovial fluid, which acts as a shock protector, preserves homeostasis, allows repair processes to be activated, and normalizes actions that affect the synthesis of endogenous cell synovial fluid, in addition to promoting greater joint mobility, reducing attrition and noise, and improving perfusion of nutrients and metabolites from the synovial fluid to vascular tissues (12). Although the benefits of using HA in association with arthrocentesis are clear in the literature, this study could have been improved by comparison with a control group of arthrocentesis alone.

Viscosupplementation with HA may be performed using one or two needles. There is a considerable difference of opinion in the current literature regarding which technique yields the best outcomes; nonetheless, the evidence shows that both techniques have high rates of clinical success (11,28-30). From this perspective, longitudinal follow-up of the patients, postoperative evaluation with MRI, and repeated application of the VAS for pain perception and MMO measurement may be important to understand tissue responses to different arthrocentesis techniques. In the long-term, these factors may influence or predict the outcome of procedures.

With regards to epidemiology, it is important to note the single-center, uncontrolled design of this study. Nevertheless, despite its geographical delimitations, the profile of the sample covered in this study was consistent with the literature in terms of sex distribution (predominantly female) and mean age (32.46±8.19 years).

Finally, one of the major benefits of arthrocentesis is its analgesic sparing effect. It is important to emphasize the chronic nature of the disease, which is caused by harmful habits and inadequate posture. Clinical follow-up and patient education can improve therapeutic success and quality of life.

## CONCLUSION

In the current study, patients with DDwoR, who were treated with arthrocentesis combined with HA injection showed a significant improvement in perceived pain and maximum mouth opening in the mid-term follow-up periods.

## AUTHOR CONTRIBUTIONS

Rossini R was responsible for the data analysis and interpretation, statistical analysis, and manuscript preparation, writing and critical revision. Grossmann E provided substantial scientific and intellectual contributions to the study conception and design, data acquisition, analysis and interpretion, technical procedures and was responsible for the approval of the final version of the manuscript. Poluha RL and Dos Santos MF performed the manuscript critical revision, and provided substantial scientific and intellectual contributions to the study. Setogutti ET performed the acquisition and interpretation of MRI scans.

## Figures and Tables

**Table 1:** Distribution of the 72 patients by categorical variables.

Variable		N (%)
Sex	Female	65 (90.3)
	Male	7 (9.7)
Side	Left	35 (48.6)
	Right	37 (51.4)
Effusion at baseline	None	1 (1.4)
	Ss	57 (79.2)
	Sb	14 (19.4)
Deflection	Left	35 (48.6)
	Right	37 (51.4)

Ss, effusion in the upper compartment; Sb, effusion in both compartments.

**Table 2:** Mean age, initial MMO, severity and duration of pain, in the 72 patients assessed in this study.

Variable	Mean (SD)
Age	32.46 (8.19)
MMO at baseline	31.35 (1.35)
Severity of pain at baseline	7.36 (1.19)
Duration of pain	9.90 (9.62)
ΔMMO (Final-Baseline)	10.72 (3.86)
ΔPain (Baseline-Final)	7.08 (1.14)

Δ, change from baseline.

MMO, maximum mouth opening; SD, standard deviation.

**Table 3:** Efficacy and tolerability of therapy.

Variable	Mean
MMO	
Baseline	31.26 [30.25-32.24]
Final	42.99 [41.28-44.23]
*p*-value	**<0.0001**

Pain	
Baseline	7.00 [6.00-8.00]
Final	0 [0-0]
*p*-value	**<0.0001**

MMO, maximum mouth opening.

**Table 4:** Correlations between pain, effusion, and MMO.

Spearman’s correlation (*p*-value)				
		Initial MMO	Final MMO	ΔMMO
Ss	Initial Pain	0.032 (0.812)	-0.068 (0.614)	-0.101 (0.453)
	Final Pain	-0.038 (0.778)	-0.387 (0.003)	-0.387 (0.003)
Sb	Initial Pain	0.338 (0.237)	-0.329 (0.251)	-0.471 (0.089)
	Final Pain	-0.055 (0.853)	-0.832 (<0.001)	-0.806 (<0.001)

Ss, effusion in the upper compartment; Sb, effusion in both compartments; MMO, maximum mouth opening.
